# Targeting Sarcopenia as an Objective Clinical Outcome in the Care of Children with Spinal Cord-Related Paralysis: A Clinician’s View

**DOI:** 10.3390/children10050837

**Published:** 2023-05-05

**Authors:** Cristina L. Sadowsky

**Affiliations:** 1 International Center for Spinal Cord Injury, Kennedy Krieger Institute, Baltimore, MD 21205, USA; sadowsky@kennedykrieger.org; 2 Department of Physical Medicine and Rehabilitation, Johns Hopkins School of Medicine, Baltimore, MD 21205, USA

**Keywords:** pediatric spinal cord injury, children, spinal cord, paralysis, function, functional outcomes, muscle mass, sarcopenia, muscle atrophy

## Abstract

Muscle loss is consistently associated with immobility and paralysis and triggers significant metabolic and functional changes. The negative effects of sarcopenia are amplified in children who are in the process of building their muscle mass as part of development. Because muscle mass loss is consistently associated with increased morbidity and mortality throughout life, optimizing the size and health of muscles following a neurologic injury is an objective target for therapeutic interventions. This review hypothesizes that muscle mass correlates with functional outcomes in children with paralysis related to spinal cord-related neurologic deficits. We propose that the measurement of muscle mass in this population can be used as an objective outcome for clinical long-term care. Finally, some practical clinical approaches to improving muscle mass are presented.

## 1. Introduction

Neuromuscular paralysis is characterized by muscle weakness and an inability to perform and/or control motor tasks. If persistent, it leads to muscle atrophy related to both neurologic injury and functional immobility. Depending on the etiology of paralysis, there are additional pathophysiologic mechanisms that can affect muscle health, like paracrine, endocrine, and metabolic changes. The muscle cell is a metabolic powerhouse with an essential role in the carbohydrate and lipid processing in the body, leading to further changes that are sure to exacerbate metabolic and functional decline [1]. Moreover, when paralysis occurs in children, when the increase in muscle mass is most dramatic (muscle mass is estimated to increase 9-fold from birth to age 10) [2], its consequences extend beyond the time of onset, affecting growth and possibly triggering neurodevelopmental motor and cognitive delay.

Skeletal muscle accounts for about 40% of body mass in adulthood [3]. Loss of skeletal muscle mass is associated with a decrease in muscle strength and/or physical performance and has been named sarcopenia [4,5]. The term sarcopenia derives from the Greek *sarx* for “flesh” and *penia* for “loss.” Primary sarcopenia occurs in aging adults when primary muscle protein breakdown outweighs protein synthesis. Neuromuscular paralysis is part of the secondary causes of muscle atrophy and sarcopenia, being related to denervation, low amount of physical activity, medication side effects, endocrine changes, and other disease-related mechanisms [6].

It is hard to comment on the incidence and prevalence of sarcopenia in children because of poorly defined age-adjusted skeletal muscle mass norms [7], but the presence of decreased muscle mass and/or muscle atrophy is almost ubiquitous in individuals with paralysis, especially paralysis related to spinal cord dysfunction [8,9]. In addition, sarcopenic obesity, a term initially described in adults [10], is quite prevalent in individuals with neurologic consequences from a spinal cord injury (SCI) [11] and is estimated to account for significant metabolic consequences [12].

**Figure 1:**
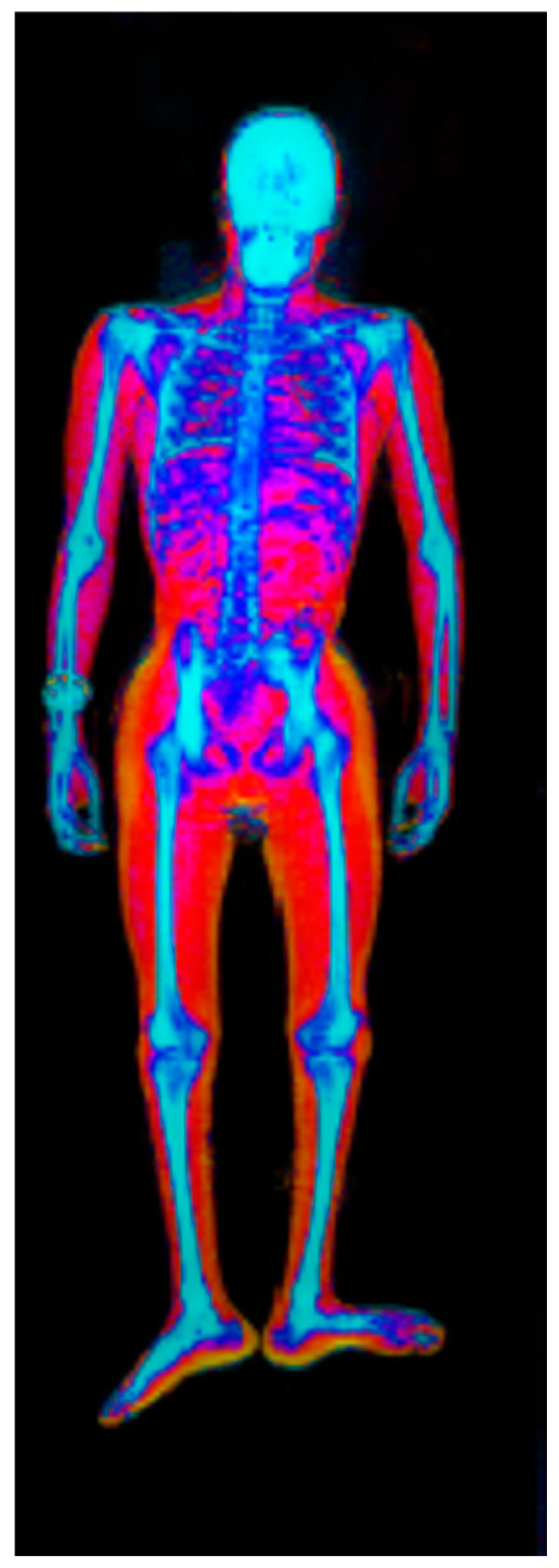
Body composition by DXA.

A first step in addressing sarcopenia is objectively measuring the amount of existing muscle. There are multiple ways to measure muscle mass [13]:

- Simple measurements and indices: anthropometrics [14], skin fold (SF), body mass index (BMI), and waist circumference (WC)
- Predictive techniques: skinfold, bio-electric impedance analysis (BIA) [15]; ultrasound [16], creatine excretion [17], total or partial body potassium per fat-free soft tissue [18], and neutron activation [19]
- Two-component techniques and models: dual-energy x-ray absorptiometry (DXA) (Figure 1) [20,21], air displacement plethysmography [22,23,24], deuterium dilution [25], computed tomography (CT) [21,26], and magnetic resonance imaging (MRI) [27,28].

While in adults, Baumgartner et al. [29] proposed using Dual-energy X-ray absorptiometry (DXA) and bioelectrical impedance analysis (BIA) as standardized methods to determine lean body mass, comparing values obtained to a normal referenced population (gender-specific young adult), no such methods are uniformly adopted for children because of ongoing changes in body composition during childhood [15].

When evaluating the muscle mass in children, each method has its own advantages and disadvantages (Table 1); the anthropometrics are easy to collect, but SF does not have age-related references, BMI does not distinguish between lean muscle and fat mass, and WC does not predict muscle mass in children [30]. The predictive and two-component methods are not always easily accessible and also incorporate assumptions that are not always proven as related to the developing child [13]. Furthermore, very little work is done when assessing muscle mass and sarcopenia in children with paralysis. 

**Figure 2:**
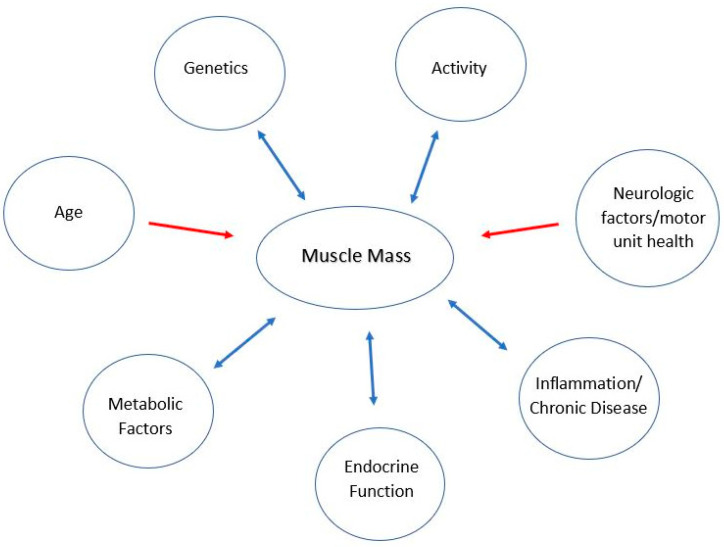
Factors affecting muscle mass.

Next, recognizing and assessing the contribution of different risk factors associated with sarcopenia is also important. There are several well-accepted contributors to muscle mass and health (Figure 2).

- Age
- Activity
- Endocrine function
- Genetics
- Inflammation and chronic conditions
- Metabolic factors
- Neurologic factors/motor unit health

All the risk factors for sarcopenia (i.e., lack of activity or lack of exercise, hormone and cytokine imbalance, abnormal protein synthesis, motor unit health, etc.) are present in children with neuromuscular paralysis of different etiologies. What complicates the work on sarcopenia in children with neuromuscular paralysis is the abnormal muscle strength and physical performance related to the disease itself, occurring in addition to the changes related to immobility. Sarcopenia, in these conditions, appears to be a self-fulfilling prophecy, a catch-22: it can be triggered by disability and leads to more disability; it increases morbidity and mortality, and the presence of morbidity/paralysis leads to sarcopenia.

### 1.1. Age and Muscle Mass

Muscle growth is especially rapid in the first two decades after birth. While pre-birth muscle mass increase is due to hyperplasia, post-natal muscle mass increase occurs because of hypertrophy [31], with an established quadratic relationship between age, muscle area, and muscle fiber density [32]; muscle cross-sectional area and mean muscle fiber size doubles between age 5 and 20, while the increase in fiber density is negligible or of a much lesser amplitude later [32]. In addition, there is a high level of adaptive capability in the muscle fiber morphology [32], which enhances the functional status of the muscle. Thus, any pathology that affects muscle development in childhood is expected to leave significant marks on further changes and function in adulthood. 

### 1.2. Activity and Muscle Mass

Following genetics, the next significant influencing factor in the development and function of muscle mass is activity. This statement is valid throughout life, not only confined to childhood. The muscle is a very plastic organ, adapting to use, diet, vascular supply, and other metabolic conditions; the adaptation occurs in response to both increased and decreased activity levels [33]. Adaptation to increased activity level occurs from either increasing the number of exerted contractions or increasing the load against which the muscle contracts. The mechanisms underlining the muscular change will be different, of course. The adaptation to increased load/resistance requires the activation of satellite cells, dormant adult stem cells located below the basal lamina that are responding to either load or injury [34]. The activated satellite cells divide in response to the specific trigger for several cycles, then further differentiate in active adult muscle cells (myocytes) or drop back to their quiescent state to be available for the next challenge. The adaptation to increasing the number of repetitive contractions is characterized by metabolic changes in the muscle fiber, specifically an increase in its oxidative capabilities [35] (mediated by mitochondria) and an increase in the microcirculation surrounding the activated muscle cell [36]. Plainly stated, strengthening exercises increase the number of myofibrils in the muscle cells, and endurance exercises improve the efficiency of the muscle. Consequently, inactivity affects both the morphology and function of the muscle cell, shortening the length of muscle cells related to sarcomere subtraction [37], decreasing mitochondrial content and function [38], and altering muscle microcirculation [39].

### 1.3. Endocrine Function and Muscle Mass

There are several hormones that play an essential role in maintaining or improving muscle mass; they include anabolic steroids (i.e., testosterone T), growth hormone (GH) and insulin-like growth factor-1 (IGF-1), and thyroid hormones [40]. 

Testosterone stimulates muscle hypertrophy by engaging multiple myogenic pathways, among which are increased protein synthesis, recruitment of satellite cells (muscle stem cells), promotion of myonuclear accretion, and pluripotent precursor cells’ commitment to developing into myotubes. Both type I and II muscle fibers are susceptible to testosterone’s hypertrophic action, but it appears that type I hypertrophy exceeds that of type II [41].

Growth hormone (GH) induces longitudinal musculoskeletal system growth. The increase in muscle mass is related to increased protein synthesis and a possible decreased rate of protein oxidation [42]. Effectively, GH stimulates whole-body protein accretion, not necessarily targeted muscle growth [43,44]. In addition, GH can mediate its effects on the muscle cell via both circulating insulin-like growth factor 1 (IGF1) and locally produced IGF1 [45].

The mechanism of action by which thyroid hormones affect muscle mass is not clearly understood, but even subclinical hypothyroidism can present with decreased muscle strength and cross-sectional area [46,47]. It is postulated that thyroid hormones participate in muscle development through protein synthesis [46]. 

### 1.4. Genetics Factors and Muscle Mass

Myogenesis takes two distinct forms—the embryonic stage, where precursors cells are derived from mesodermic structures [48] and replicate, thus forming the baseline template of the individuals’ musculoskeletal striated muscle bulk, and the post-natal stage, mostly triggered by cell damage and involving activation of quiescent satellite cells. 

Both muscles and bones grow throughout childhood and early adulthood, achieving peak mass in the second to third decade [32]. It is generally accepted that the performance of a muscle is related to the number and types of fibers it is composed of [3]. A baseline number of muscle fibers is relatively set at birth as a result of embryonic myogenesis [49], thus being dependent on genetics and pre-natal factors. Heritability [50] and gene polymorphism [51], maternal nutrition [52,53,54], and weight at birth [55,56,57,58] are factors that have been found to influence myogenesis. 

### 1.5. Inflammation and Chronic Conditions and Muscle Mass

Muscle atrophy related to inactivity and paralysis is a result of both decreased protein synthesis and increased protein lysis, and oxidative stress has been identified as an important modulator in cell signaling pathways [59]. Experimental models of inactivity reveal that protein synthesis is markedly reduced at 48 h post immobilization [60]. The increased proteolysis associated with lack of activity is subsequently related to mitochondrial dysfunction [61]. In addition, the loss of muscle mass associated with chronic conditions like muscular dystrophy and denervation has also been shown to be linked to a form of apoptosis called “myonuclear apoptosis” [62,63]. Possible mechanisms responsible for myonuclear apoptosis are caspase-related and mitochondrial and receptor-mediated programmed cell death [59].

Pro-inflammatory cytokines, among which are interleukin (IL)-1, 6, tumor necrosis factor-alpha (TNF-α), interferon-alpha (IFN-α), and gamma (IFN-γ), can activate the hypothalamic-pituitary-adrenal axis, increasing stress-hormone production which, in turn, can inhibit muscle growth through triggering insulin and growth-factor resistance [64,65]. Another argument supporting the role of inflammation in muscle mass health is gleaned from administering lactobacilli, a common modulator of inflammation in healthy individuals, to mice with cancer cachexia, with the resulting reversal of muscle atrophy [66,67].

Larger doses of non-steroidal anti-inflammatory drugs (NSAIDs), which target inflammation through the cyclooxygenase (COX) enzyme, have also been found to downregulate protein synthesis following muscle injury, thus impairing muscle repair and hypertrophy [68].

### 1.6. Metabolic Factors and Muscle Mass

Nutrition and, particularly, protein intake play essential roles in determining the muscle mass phenotype both in the pre-natal and post-natal periods. There is animal model evidence for the role of maternal nutrition in fetal programming of the amount of muscle cell production during organogenesis [69], with maternal undernutrition negatively impacting the number of muscle fibers formed and overnutrition committing mesenchymal cells toward adipocytes [70]. In addition, because muscle mass health depends on an ongoing balanced cycle of protein synthesis and degradation [71], an adequate nutritional intake of amino acid-supplying proteins constitutes a staple of muscle mass well-being.

Vitamin D’s essential role in muscle mass regulation has been more intensely studied in the past decade. The so-called “genomic effect” of vitamin D (mediated through vitamin D receptors VDR) is important in gene transcription, but vitamin D also appears to improve progenitor muscle cell migration to injured sites and modulates the intracellular calcium entry, thus influencing the contractile abilities of the muscle fibers [72]. 

The interactions between muscle mass and metabolism are extensive. Aside from the pre-natal balance between myocytes and adipocyte formation, the post-natal amount and quality of muscle mass play an indubitable role in lifelong metabolism. It has been shown that muscle strength is inversely correlated with the prevalence of dyslipidemia [73]. Moreover, there are numerous studies showing the protective effect of muscle strength on cardiometabolic factors in general in both children and young adults [74,75]. Sarcopenic obesity, a term indicating the coexistence of obesity and low muscle mass, has been shown to have significant negative cardiometabolic outcomes, worsen non-alcoholic fatty liver disease (NAFLD) severity, increase inflammation, and impair mental health [76].

In adults with SCI, studies have shown that bigger muscles are capable of losing more muscle mass [8]. The big muscles are the sites of glucose metabolism; thus, the decrease in muscle mass following SCI is expected to contribute significantly to glucose metabolism dysfunction [77].

### 1.7. Neurologic Factors/Motor Unit Health and Muscle Mass

Both muscle anatomy and function are dependent on adequate neurologic function. The muscle-fiber contraction is triggered by the transmission of an electric potential from a healthy motor neuron located in the anterior horn of the spinal cord. The motor neuron itself is activated by the motor cortex, which sends descending electrical signals through the spinal cord. Activation of the motor neuron triggers the release of the neurotransmitter acetylcholine at the neuromuscular junction, with subsequent initiation of muscle-fiber contraction. Muscle fibers recruitment is progressive, with small muscle fibers recruited first, triggered by low force, and additional large fibers being recruited later as the demand for stronger contractions emerges [78]. As the ability to maximally recruit motor units depends on the maturity and integrity of the central nervous system (CNS) and the musculoskeletal system, children are expected to be able to generate lower muscle forces even when the amount of muscle strength is adjusted to the muscle size [79]. Denervated muscle fibers undergo cellular and molecular changes, including the development of extra-junctional acetylcholine receptors and tetrodotoxin-resistant action potentials [80]. The ability to optimally contract the muscle against a mechanical load affects the rate of protein synthesis, thus contributing to muscle mass health [81].

## 2. Muscle Mass-Function Connection

The fact that muscle mass is a strong predictor of health and performance is hard to oversee. Muscle mass has been shown to affect cognitive function [82] and daily function [83] and is essential for general health and the prevention of disease [84].

Lean muscle mass has been correlated with strength and functional performance in healthy children [55,85,86,87] and in those with chronic, long-standing conditions [88,89].

Because standardized measures of muscle strength have only been developed for children older than four years of age [90], muscle mass is frequently used as a surrogate for muscle strength in the pediatric population, especially in young children where the ability to follow directions, motivation, and ability to sustain an ongoing effort is variable. 

## 3. Studies of Muscle Mass in Paralysis Related to Spinal Cord Disease

Studies that look at muscle mass and its correlation with function (neurologic and day-to-day), as well as cardio-metabolic changes in pediatric populations with spinal cord-related paralysis, are few and far between. In fact, a literature search conducted in five databases, Web of Science, PubMed, CINAHL, Cochrane, and EMBASE, only yielded six prospective and/or retrospective cross-sectional cohort studies and one review paper that addressed this topic specifically (Table 2). Given the tight relationship between muscle mass and function and the significant loss of function associated with paralysis, evaluating factors that can improve or restore muscle mass in the context of paralysis appears worthy of further research. Muscle mass restoration in individuals with paralysis is not easy, but not impossible either, and concentrating clinical efforts to accomplish such an objective outcome aligns with the goals of intervention in the field of physiatry and rehabilitation. 

In 2007, Liusuwan et al. [91] assessed the effects of a 16-week nutrition education and exercise program on the health and fitness of 14 adolescents with mobility impairment due to spinal cord-related paralysis from myelomeningocele and SCI and found that there was a significant increase in whole-body lean tissue, which functionally translated into an increase in maximum power output and work efficiency; the program also increased muscle strength in proximal shoulder muscles. 

In another paper in the same year, 2007, Liusuwan et al. [92] showed that children 11–21 years of age with paralysis related to SCI (n = 33) or myelomeningocele (n = 66) had significant lean tissue mass deficits as determined by DXA when compared to able-bodied controls; they also showed significantly lower resting energy expenditure associated with the decreased total lean muscle mass. 

Liu et al. [93], in a retrospective case series, documented the changes in lean muscle and bone mass following the onset of both traumatic and non-traumatic spinal cord-related paralysis in 18 children seen at one single center in Australia between 1990 and 2000. Children were an average of 5.3 (0.5–15.6) years old at paralysis onset, and the mean follow-up was 5.0 ± 3.6 years (range 0.4–12.4 years) following the onset of neurologic deficit. There was a group of eight children that had their initial DXA bone and lean muscle mass assessed within 0.3 years of paralysis onset and thorough follow-up DXA done 1.2 years later (0.8–1.3), it was found that most of the lower limbs muscle mass loss was in the first year post-injury. This is, of course, important as it sets up the timing for interventions meant to limit muscle mass loss. In the same paper, it was apparent that muscle (and bone) mass showed age-appropriate accrual starting year 2 post-onset of paralysis. 

In 2011, Johnston et al. [94] performed a prospective randomized controlled study on 30 children with motor complete and incomplete tetraplegia and paraplegia following an SCI aiming to determine the effect of passive cycling, electrical stimulation (e-stim), and e-stim assisted cycling on thigh muscle volume and stimulated muscle strength. Children performed the intervention in the home setting for six months (1 h of exercise three times/week), and muscle volume (determined with an MRI) and electrically stimulated isometric muscle strength (determined with a computerized dynamometer) were recorded before and after the intervention. Twenty-four children (eight from each intervention group) had muscle volume data, and 27 (nine from each group) had stimulated muscle strength data, which showed that the muscles that increased in volume as a consequence of the intervention had also increased muscle strength. 

Biggin et al. [95], in a retrospective cross-sectional cohort study of 19 pediatric patients with SCI (nine with paraplegia and 10 with tetraplegia), showed that individuals that were able to stand had greater calf muscle cross-sectional area as determined by peripheral quantitative computed tomography (pQCT).

Finally, in 2022, Curley et al. [96] analyzed the relationship between muscle mass (determined using DXA) and function (determined using Physical Abilities and Mobility Scale PAMS) in a retrospective analysis of 41 children with acute flaccid myelitis (AFM) and showed that lean muscle mass correlated with bone mass and functional performance as assessed with PAMS. Interestingly enough, lean muscle mass did not correlate with muscle strength in this cohort of children with AFM. 

## 4. Sarcopenia Reversal and Other Strategies to Increase Muscle Mass

### 4.1. Exercise

Many studies have shown that physical activity plays an essential role in preserving and even restoring muscle mass.

The current Centers for Disease Control (CDC) recommendations for the amount of physical activity children need depends on age, while the recommendation for children ages three through five years is vaguer, with CDC recommending that children aged three to five need to be active throughout the day, the recommendation for children and adolescents aged six through 17 is for moderate-to-vigorous intensity physical activity for 60 min each day [97]. Healthy Children 2030 program operating out of the Department of Health and Human Services, estimated that only 25.9 percent of children aged six to thirteen years met the current aerobic physical activity guideline in 2016–2017; this number dropped to 23.6 percent in 2020–2021, likely as a consequence of the pandemic [98]. Children with paralysis have even more activity limitations, leading to increased risks for the development of cardio-metabolic comorbidities and death at a higher rate than able-bodied children [99].

Both strength and resistance training have been consistently shown to positively affect muscle mass and strength in children and adults, although resistance training is more associated with increased muscle mass [100,101]. The notion that resistance training in a growing child is harmful by stunting growth and damaging epiphyseal plates or is not effective because of a lack of adequate hormonal milieu was disproven a long time ago [102]. While muscle hypertrophy occurs less in children than adults following resistance training [103], the net effect of resistance training in children with and without disabilities is an improvement in function and performance [104,105] based mostly on neuro-modulation and muscle fiber activation [106]. Considering the fact that paralysis specifically affects this ability to activate the motor effector, it appears evident that resistance and strength training should play a major role in restoring function in this population. Traditional compensatory rehabilitation in children with paralysis was focused on achieving functional tasks utilizing mostly residual motor function. In the past 20 years, the emergence of activity-based restorative therapies (ABRT) [107] has been targeting activation of the muscles above and below the neurologic injury level and, by employing highly repetitive muscle patterns activations in the context of muscle paralysis, effectively functioning as a regimen of resistance training.

Resistance training in itself induces significant endogenous hormonal (testosterone, growth hormone, insulin growth factor) elevations [108], and, in turn, these hormones play a major role in muscle health and mass [40]. In children, especially adolescents and young adults with paralysis, enhancing the effect of exercise by using advanced techniques like low load resistive training coupled with blood flow restriction and modified accentuated eccentric loading training [100] can potentially maximize the result on the recruited muscle fibers. In order to improve the recruitment of muscle fibers, the utilization of electrical stimulation (e-stim) has been consistently shown to be effective and efficient [109]. E-stim-assisted resistance training has been shown to objectively improve muscle mass in adults with SCI [110], and percutaneous electrical stimulation has been proven to strengthen and improve the size of the partially denervated quadriceps following SCI in young adults [111]. Functional electrical stimulation (FES) has also been shown to improve muscle strength and mass in adults with SCI [112], and its use was deemed safe in children with various disabilities, including those with spinal cord-related paralysis [113]. 

### 4.2. Diet

When considering sarcopenia reversal, diet also plays a major role. 

*Protein* intake, as previously mentioned, plays a significant role in muscle health in the pediatric population. According to the Protein-Stat model for the control of growth, a minimum intake of protein constitutes the “anabolic drive” that regulates bone growth which, in turn, allows for muscle myofiber growth. However, excessive early-life protein intake is associated with obesity or a propensity to obesity later in life [114,115]. The current Dietary Reference Intakes for protein indicate that able-bodied children aged 4–13 years and 14–18 years require 0.95 and 0.85 g·kg^−1^·day^−1^, respectively [116]; these values are not adjusted for the amount of physical activity exerted. There are no specific studies assessing protein intake in children with spinal cord-related paralysis. In general, the resting metabolism in adult individuals with SCI has been assessed to be 14–27% lower than in their able-bodied counterparts [117]. In malnourished children with cerebral palsy, an intake of 2.0 g/kg per day of protein has been proposed to promote “catch-up” growth [118]. The information derived from the existing literature appears to suggest that increasing the amount of protein intake over daily recommended values will not induce additional muscle growth over the genotypically determined muscle mass [114]. Nevertheless, in the context of addressing SCI-related sarcopenia in a developing child with paralysis, a careful assessment of needs pertaining to muscle loss and physical activity, and nutritional protein intake is highly warranted.

*Creatine monohydrate* has been documented to have anabolic potential and has been used in conjunction with exercise to improve muscle strength and volume [119,120]. In adults with spinal cord injuries, a daily creatine dose between 3–20 g has been shown to improve upper body stress and work capacity [121,122]. Studies of creatine supplementation in able-bodied children and adolescents (up to 30 g/day) showed consistent improvement in motor tasks performance and no significant side effects, while the studies looking at creatine supplementation (0.75–12 g/day) in children with different pathologies, including muscular dystrophies showed variable changes, from temporary prevention of muscle atrophy to small increases in muscle fiber thickness [123].

Nicotinamide riboside (NR), a nicotinamide adenine dinucleotide (NAD+) precursor, is an important coenzyme in major anabolic and catabolic reactions throughout life. In animal models, oral supplementation with NR has been shown to improve mitochondrial function [124] and the regenerative potential of stem cells [125] and positively affect numerous neuro-degenerative and neuroinflammatory pathways [126]. The supplement has not been associated with significant side effects, and currently, there are numerous clinical trials attempting to assess efficacy in sarcopenia and other neurodegenerative diseases, including multiple sclerosis, neuropathies, and amyotrophic lateral sclerosis (ALS) [127] in both adults and adolescents [128].

## 5. Summary

Sarcopenia is immediate and profound following spinal cord injury-related paralysis. The diminished muscle mass negatively affects the development and metabolism of the growing child and is associated with increased morbidity and mortality [129]. There are well-described, safe, and effective interventions to address sarcopenia in children with paralysis, and optimizing and restoring muscle mass should be a consistent and well-defined intervention when managing post-paralysis deficits. Assessing and monitoring muscle mass can be done clinically, and simple, noninvasive methods are available. Consistent and ongoing evaluation of muscle mass can be a clear, objective outcome to be measured in the health maintenance of children with spinal cord-related paralysis. 

## Figures and Tables

**Table 1:** Muscle mass measurement methods.

Method	Cost	Availability	Pros	Cons
Anthropometrics (SF, BMI, WC)	+	++++	Noninvasive Can be used for screening	Poor precision in obese Some operator training required
BIA	++	++	Easy to use Reproducible	Subject dependent (hydration status, recent food intake, body/air temperature, recent physical activity status)
US	++	+++	Safe Noninvasive Radiation free	Operator dependent technique Muscle- fat interface hard to determine
Biochemical markers (K, Cr)	++	++	Safe	Requires appropriate 24-h urine collection
DXA	++	++	Safe Can provide regional estimates (trunk, legs, arms)	Center-based (not portable) Subject size limitations Variations between manufacturer’s software do not allow for comparisons Water-bone free interface hard to determine
CT	++++	++	Highly reliable	Radiation exposure Center-based
MRI	++++	++	Highly reliable No radiation involved	Center-based Variations between manufacturer’s software do not allow for comparisons Subject size limitations

+ = least, ++++ = most.

**Table 2:** Studies assessing muscle-mass function in children with SCI.

Author, Year, Study Design	Sample, Methods	Results	Conclusions
Liusuwan 2007 [91], USA Prospective study with intervention	*N* = 20 adolescents with spinal cord dysfunction (SCD) related to spinal cord injury or spina bifida (7 females, length of injury: 2 18 years from injury onset) Age: 11–18 years (mean 15.4 ± 2.2 years) Intervention: 16 weeks program consisting of exercise, education, and behavioral modification for nutritional intake Lean muscle body mass measured by DXA	*N* = 14 adolescents completed the study Whole body lean tissue and muscle work efficiency increased; some muscle strength (shoulder extensors) also increased.	A multidisciplinary program involving exercise, education, and nutrition can increase function and lean muscle body mass in adolescents with paralysis related to SCD
Liusuwan, 2007 [92], USA Retrospective cross-sectional study, no intervention	*N* = 215 children Age: 11–21 33 with SCI (12 females), 66 with spinal bifida SB (30 females), 31 overweight able body (12 females), 85 able body controls with normal BMI (44 females) Lean muscle body mass measured by DXA	Gender differences were observed in all groups; SCI and SB adolescents had significantly lower lean muscle body mass than able body ones (controls and overweight) Resting energy expenditure was lower in adolescents with SCI and SB	Gender differences in muscle mass are present in children with and without paralysis. Lean muscle body mass was lower in children with SC-related paralysis Resting energy expenditure directly correlated with lean muscle body mass
Liu, 2008 [93], Australia Retrospective cross-sectional study, no intervention	*N* = 18 children with pediatric-onset SCI (9 females, 0.1–7.2 years from injury onset) Age: 5.3–17.1 Follow-up time: 5.0 ± 3.6 years (range 0.4–12.4 years) Lean muscle body mass measured using DXA.	Lean muscle body mass was decreased below injury level. Lean muscle body mass loss occurred mostly in the first year after onset of neurologic deficit. After first year of paralysis, age-appropriate increase in lean body muscle mass occurred	Lean body muscle mass loss occurs rapidly following neurologic injury onset in children with pediatric-onset SCI
Johnston, 2011 [94], USA Prospective randomized controlled study, with intervention	*N* = 30 children with chronic SCI (>12 months from injury onset) Age: 5–13 years Interventions: 6 months of exercise-cycling (functional electrically stimulated FESC and passive PC and electrically stimulated strengthening (ES) Muscle mass measured with MRI	ES group had greatest increase in volume of the stimulated muscle; FES group had greatest increase in strength in stimulated muscles	ES and FES increase both muscle volume and strength in children with chronic SCI
Biggin, 2013 [95], Australia Retrospective cross-sectional cohort study, no intervention	*N* = 19 children with SCI (9 females, 9 with paraplegia and 10 with tetraplegia). Age: 1.9–18.8 Muscle mass measured by Cross-sectional computer tomography pQCT	Children that had the ability to stand had greater cross-sectional calf muscle area compared with those unable to stand.	Standing helps preserve calf muscle mass
Curley, 2022 [96], USA Retrospective cross-sectional study, no intervention	*N* = 41 children with acute flaccid myelitis (AFM) (24 females, time from injury onset 3–57 months) Age: 4 months–21 years old Lean muscle body mass measured using DXA	Functional performance, as assessed by the validated functional score Physical Abilities and Mobility Scale (PAMS), directly correlates with lean muscle mass as measured by DXA but not with muscle strength in children with AFM	Lean muscle mass correlates with functional performance
